# Optimizing Fixation and Biologic Augmentation in Aseptic Femoral Nonunion Management: A Retrospective Case Series

**DOI:** 10.7759/cureus.66343

**Published:** 2024-08-06

**Authors:** Sarthak Walia, Abhay Agarwal, Ishan Shevate, Rahul Salunkhe, Rishabh Aggarwal

**Affiliations:** 1 Orthopedics, Dr. D. Y. Patil Medical College, Hospital and Research Centre, Pune, IND; 2 Orthopedics, Apollo Excelcare Hospital, Guwahati, IND

**Keywords:** fracture nonunion, orthopedic trauma, hardware removal, fracture revision, fracture healing, broken hardware

## Abstract

Nonunion poses significant difficulties for both patients and orthopedic surgeons, often requiring intricate reconstructive surgeries to achieve bone healing and eliminate infections. Surgeons must navigate numerous contributing factors to nonunion, and they also face challenging hardware issues during revision procedures. These issues can include infections, loose or failing hardware, misaligned components, or inappropriate hardware configurations. This case series includes five cases of nonunion femur fractures and the goal is to carefully analyze the best treatment option for treating nonunion. All the cases underwent the removal of whole or part of the hardware followed by bone grafting and attainment of the stable construct with load-sharing devices and augmentation with neutralizing plates. All the cases had a radiological bone union at an average of four to seven months with improvement of Harris Hip Score.

## Introduction

High-energy trauma still results in nonunion despite advancements in trauma care, better surgical methods, state-of-the-art implants, and the development of novel adjuvants to healing, biologic agents. Significant socioeconomic issues, including prolonged morbidity, the inability to regain full function, abnormal gait, the need for reoperations, and psycho-emotional impairment, arise for patients with nonunion of the femoral shaft. Moreover, it presents a therapeutic challenge for orthopedic surgeons, who need to take into account a variety of treatment approaches, deformity correction, infection management, and quick patient recovery [1].

The high-energy injuries frequently result in open fractures or complicated fracture configurations accompanied by substantial soft tissue injury and bone fragmentation, making them particularly difficult to treat due to bone gaps, instability, and a high risk of infection, requiring multiple surgeries [2,3]. Although intramedullary nailing (IMN) has excellent clinical outcomes for treating femur fractures, it is not without risks, including nail or locking screw breakage and a nonunion rate ranging from 0.5% to 10% [4]. In nonunion cases, achieving stable fixation and fracture union through exchange nailing alone can be difficult, because the nail may not fit the wide distal canal adequately to prevent bending and torsional forces, limiting secure fixation and mechanical stability [5-8]. While plates provide strength against torsional forces, they are less effective against bending and axial forces, particularly when medial bone support is absent, as is common in revision femur fractures [9]. Combining the strengths of both methods resulted in the concept of plate augmentation in conjunction with the nail in situ for nonunions, which provides additional stability while also promoting the union of fracture [10-13].

The main aim of this case series is to evaluate the outcomes of multiple nonunited femoral fractures treated with IMN, augmentation with plate fixation, and bone grafting in a single stage.

## Case presentation

This case series was retrospectively conducted over the period from December 2022 to February 2023; five patients aged 16 to 70 with femoral shaft nonunion underwent surgery that included IMN, plate augmentation, and bone grafting. Neither of the patients demonstrated clinical signs of infection. Two of the five cases had a history of falls from heights, while three had a history of road traffic accidents (RTA) as their initial trauma. None of the patients had any known comorbidities. Two patients had a history of tobacco chewing and were heavy smokers (more than 25 cigarettes daily). Radiographically, femoral fractures were classified using the Winquist-Hansen and AO classifications, while nonunion types were classified using the Weber and Cech classifications. To precisely rule out infection, specific measures were implemented at each stage of the surgery. Prior to surgery, clinical examinations and lab tests were performed, including infective markers. During the surgical procedure, we inspected for any signs of purulent discharge and obtained at least five tissue samples for HPE examination, culture, and sensitivity testing.

Surgical technique

All the patients underwent surgical intervention after pre-anesthesia clearance and informed written consent. The first step of the intervention involved preparing the medulla for antegrade IMN. Once the reaming was done to the appropriate diameter, a nail one size smaller was inserted into the medulla. The objective of this initial nail insertion was to maintain alignment in the coronal and sagittal plane, allowing the scope for further rotational alignment adjustments. The bone gap and defect were then calculated with a ruler to decide whether to sustain bone length utilizing an autogenous cortico-cancellous iliac bone graft or to accept slight shortening, usually less than 1 cm.

Depending on the fracture gap, we placed a broad dynamic compression plate (DCP) on the femur in the second step. The plate screw holes were spaced obliquely, allowing screws to be inserted without hitting the nail and, if feasible, allowing fixation with bicortical screws. The dynamic compression principle of the plate allowed for axial compression at the fracture site. Both proximally and distally, at least eight cortices were fixed to the fracture site, with proximal fixation occurring first. Distal fixation was done after correcting distal fragment rotation under fluoroscopic guidance.

The bone graft was placed at the fracture site after decortication to promote bone healing. The suction drain was put below the subcutaneous layer and the wound was closed in layers.

Postoperative management

**Figure 1:**
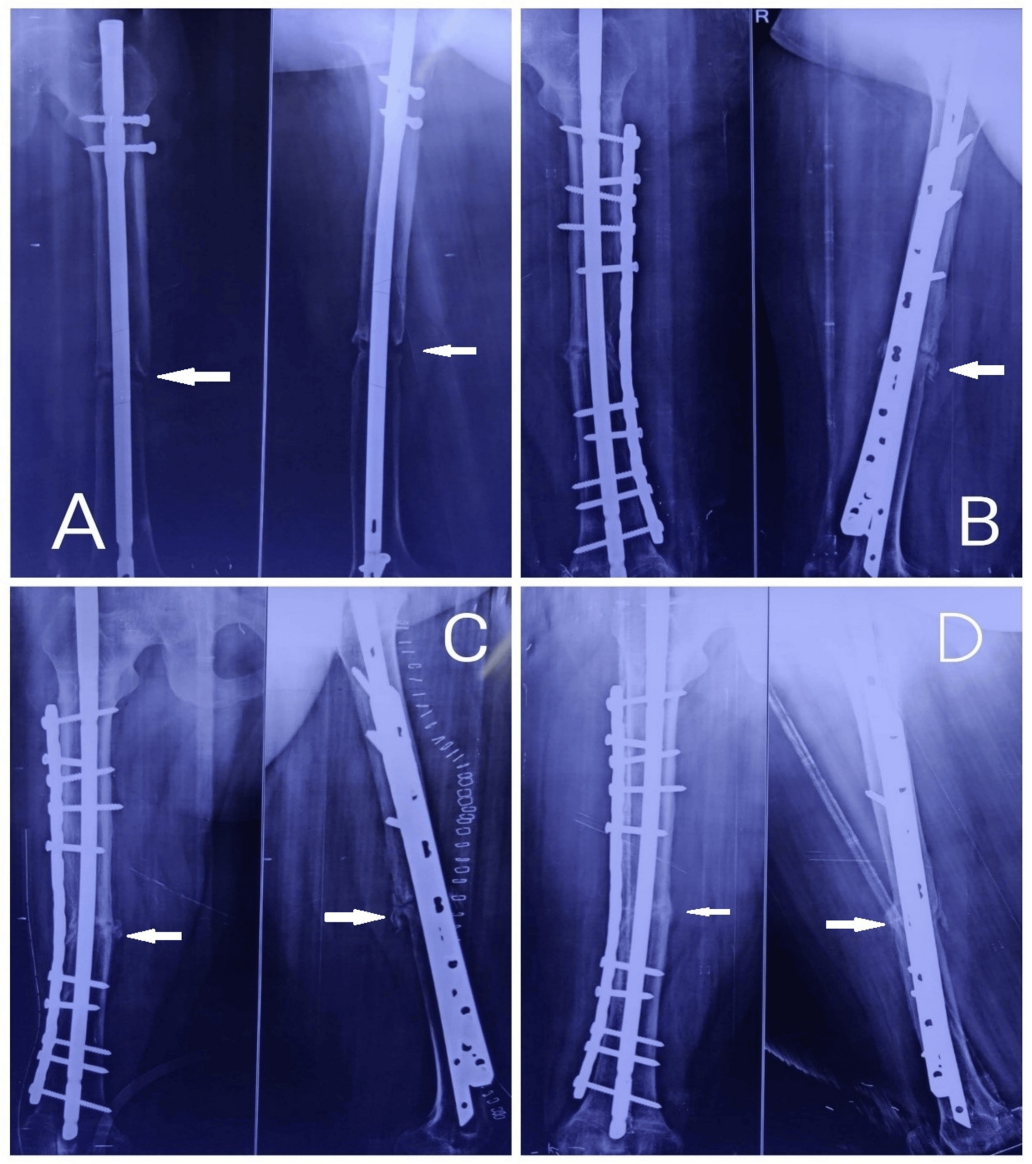
A 63-year-old female with right femur shaft fracture nonunion. A. Preoperative radiograph taken 10 months after initial surgery showing nonunion at the fracture site. B. Immediate postoperative radiograph following dynamization of the existing nail with DCP plate and bone graft augmentation. C. Six weeks after surgery, the radiograph shows intact implants and signs of bone union. D. Three months postoperative radiograph showing bone union with good consolidation at the fracture site. DCP, dynamic compression plate

**Figure 2:**
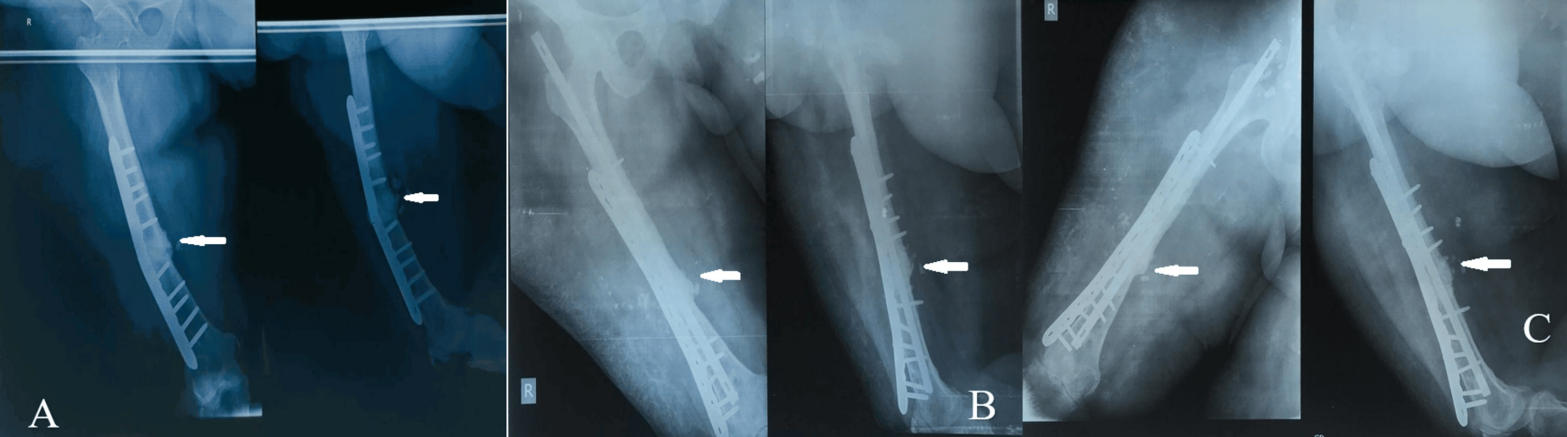
A 36-year-old male with implant breakage and nonunion of the right femur shaft fracture. A. Preoperative radiograph taken nine months after initial surgery revealing implant breakage and nonunion. B. Immediate postoperative radiograph following IMN, DCP plate, and graft augmentation. C. Three months postoperative radiograph displaying bone union with robust consolidation at the fracture site in a radiograph taken three months postoperatively. IMN, intramedullary nailing; DCP, dynamic compression plate

**Figure 3:**
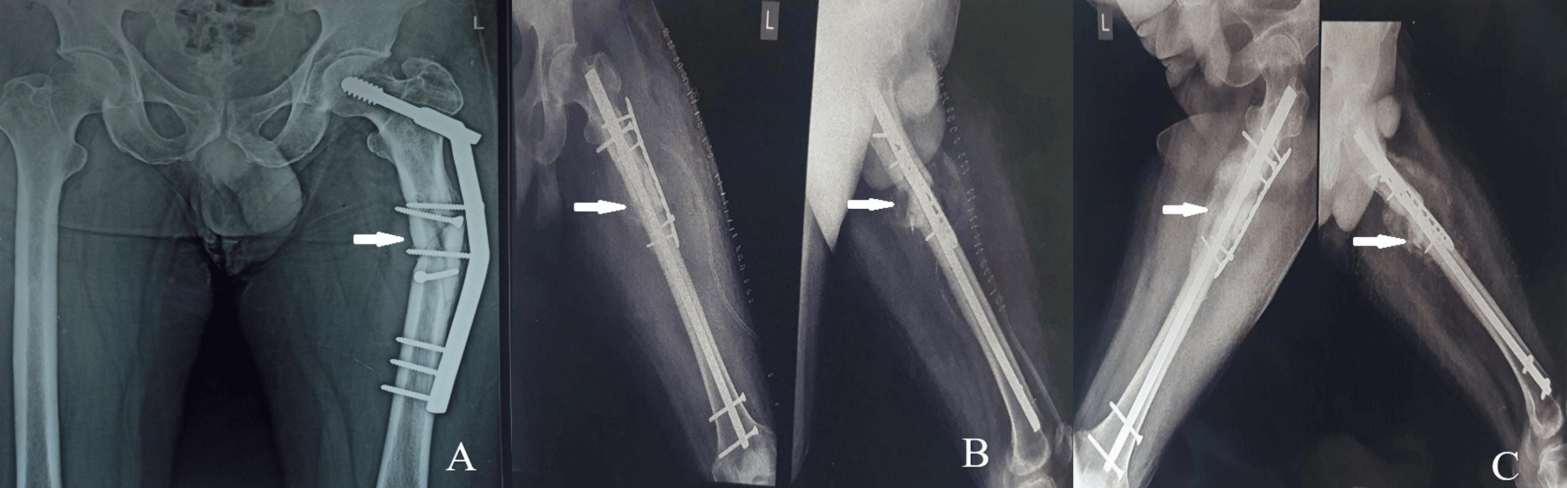
A 54-year-old male with implant failure and nonunion of left femur proximal 1/3rd shaft fracture repaired with a dynamic hip screw. A. Preoperative radiograph taken nine months after dynamic hip screw fixation, showing implant failure and nonunion. B. Immediate postoperative radiograph following IMN with DCP plate and graft augmentation. C. A radiograph taken three months postsurgery demonstrates bone union with satisfactory consolidation observed at the fracture site. IMN, intramedullary nailing; DCP, dynamic compression plate

All patients were given proper antibiotic coverage until discharge. Full weight bearing was delayed till bone union was visible on plain radiographs. Patients’ knee range of motion was started as per tolerance and were motivated to quit smoking. Clinical and radiological follow-ups were done at 14 days (for stitch removal) followed by six weeks, three months, and one year. Preoperative, postoperative, and follow-up radiographs are shown in Figures 1-3.

## Discussion

Intramedullary nails are extensively employed for femoral shaft fractures because of their high success rate in achieving bone union [14]. Exchange nailing has traditionally been the preferred treatment for femoral shaft nonunions subsequent to IMN [15,16]. Compared to exchange nailing, plate augmentation has demonstrated better outcomes and fewer complications [17]. In this case series, plating along with iliac bone grafting was used in conjunction with IMN in all cases. A 100% union rate was achieved in an average of 4.9 months (range: four to seven months), with improvements in Harris Hip scores. There were no complications with this technique.

The study has several limitations. First of all, it is retrospective, which can lead to biases and limitations in data collection and analysis. Second, the lack of a control group makes it difficult to compare outcomes with alternative treatment approaches or establish causality definitively. Finally, the small number of cases in this study may limit the findings' generalizability and ability to detect rare complications or outcomes.

The union rate observed in our study is consistent with findings reported in other studies in which plate augmentation with bone grafting was done. For example, in a retrospective study by Jhunjhunwala and Dhawale in which 40 patients with nonunion of the femoral shaft were included and underwent treatment with an interlocking intramedullary nail, plate augmentation resulted in a union rate of 97.5% [18]. In their study, 24 patients with oligotrophic nonunion underwent autogenous iliac bone grafting. In addition, nine patients had their nails exchanged for larger sizes. However, the study did not specify the criteria used to select patients for each procedure.

Exchange nailing has long been the preferred method for treating femoral shaft nonunions [17]. However, it can be difficult, especially in cases where there are broken implants or heterotopic calcification at the entry site. Exchange nailing failure has been documented in nonunions of long bones that are associated with fragmented fractures, bone deficiencies, and fractures situated at the junction between the metaphysis and diaphysis.

The use of an iliac bone graft is thought to be critical to the success of the plate augmentation technique [19,20]. Prior research has underscored the importance of biological supplementation with iliac bone grafts, employed in all instances of atrophic nonunion and the majority of hypertrophic nonunion cases [19,20]. Irrespective of the defect's size, the consensus among most authors is to utilize autogenous iliac cortico-cancellous bone grafts [18,19].

## Conclusions

In the authors' experience, although nonunions are difficult to treat, they can be dealt with near certainty to ensure good bone healing. Proper preoperative planning plays an important role in anticipating various potential outcomes. Multiple implants and bone grafting techniques can be used to adjust for bone defects and nonunion geometry.

IMN with DCP plate augmentation and bone grafting is an effective treatment option for nonunion, with positive clinical and radiological outcomes.
